# Aeroallergen IgE-Reactivity Patterns and Respiratory Allergy in Children and Adults: A Retrospective Study in 1711 Patients from the Central Poland Urban Area

**DOI:** 10.3390/medicina61091676

**Published:** 2025-09-15

**Authors:** Barbara Majkowska-Wojciechowska, Maciej Kulma, Marcin Kurowski

**Affiliations:** Department of Immunology and Allergy, Medical University of Łódź, 90-419 Łódź, Poland

**Keywords:** aeroallergens, IgE-reactivity, allergen exposure, allergic diseases, allergy epidemiology, age differences, sex differences

## Abstract

*Background and Objectives*: Effects of allergen exposure may be modified through endogenous and exogenous factors, resulting in heterogeneity of clinical features, time course and intensity of symptoms of allergic disease. This creates challenges in allergy diagnosis and management, yet studies addressing the variability of allergen reactivity in relation to potential modifying factors are not numerous. The aim of the study was to retrospectively analyze the frequency and profile of reactivity to inhalant allergen extracts in patients followed in a single center in the years 2017–2020, in relation to sex, age, clinical symptoms and final clinical diagnosis. *Materials and Methods*: This is a retrospective analysis of skin prick test (SPT) results in 1711 outpatients, performed with dust mites, pollen (alder, hazel, birch, grasses, rye, mugwort), cat, dog and *Alternaria* allergens. Reactivity profiles were assessed in the entire population divided into sex and age subgroups. Relationships between SPT results, age, sex and clinical diagnosis were assessed using factor analysis. *Results*: The highest reactivity frequencies were found for grass (60.5%), rye (57.22%), birch (47.34%), alder (42.5%) and *Dermatophagoides pteronyssinus* mites (41.8%). Monovalent reactivity was found mainly to mugwort (3.2%, n = 55), followed by cat and mites allergens. Reactivity to ≥1 allergen was more frequent in males. The risk of allergic rhinitis (AR) was significantly higher in subjects allergic to grass, rye, hazel and alder pollen, as compared to subjects non-sensitized to these allergens. Reactivity to perennial allergens (dog, cat and dust mites) was significantly associated with asthma diagnosis. The risk of developing atopic dermatitis was significantly associated with reactivity to birch and alder pollen. *Conclusions*: SPTs are a valuable tool for assessing the occurrence of atopy and allergy. Reactivity to specific aeroallergens may be associated with increased probability of development of a given atopic condition. This warrants further studies regarding the interplay between possible modifiers of allergen exposure effects.

## 1. Introduction

Allergies are common, non-infectious, chronic diseases affecting both children [1] and adults [2]. The growing number of respiratory diseases caused by inhaled allergens is a serious challenge for healthcare. Epidemiological studies indicate that about 40–50% of the world’s population suffers from one or more allergic disease [3,4]. Among them, allergic rhinitis (AR) is most frequent and considered a global health problem. AR can co-occur with other allergic manifestations, e.g., asthma or conjunctivitis, thereby significantly influencing different aspects of the daily quality of life [5,6]. A considerable diagnostic challenge may be encountered in cases of IgE-reactivity and allergic inflammation limited to local nasal mucosa with no ascertainable IgE’s, either in skin prick tests (SPTs) or in serum [7,8,9].

Multiple factors contributing to allergy pathogenesis and clearly differentiated clinical symptoms have already been identified. Although the relationship between allergic diseases and skin test results has already been investigated [10,11], in our opinion there is a lack of in-depth, detailed analyses of the number, spectrum and variability of sensitizing allergens and their relation to the sex and age of patients referred for allergy consultations. Therefore, we have decided to assess the distribution of the patterns of sensitizing allergens in the course of different allergic conditions. This cross-sectional, single-center study aimed to analyze the frequency of reactivity to allergens and its association with the presence of physician-diagnosed allergic disease in children and adults followed in an outpatient tertiary center.

The multinational ‘GAN’ Phase I study of the prevalence of rhinoconjunctivitis, asthma and eczema in children and adolescents in 25 countries showed a persistently high burden of these conditions and large differences in their prevalence between populations from different countries [12]. One of the most important risk factors for allergic rhinitis (AR) and asthma is IgE-dependent reactivity to one or more allergens [13].

Polish studies conducted in nine selected regions of the country indicate that the frequency of physician-diagnosed AR is 28.9%. The frequency of clinically confirmed asthma was 11.4% in children and 9.5% in adults [14]. In our previous study from Łódź and the Łódź Region (Central Poland), it was found that positive skin test results with at least one allergen from the standard airborne allergens panel were found in as many as 63.7% of urban children and 22.7% of children living in rural areas. At least one allergic disease was diagnosed in as many as 43.3% of urban children and 12.8% of rural children. Potential risk factors associated with the development of allergy differed significantly between urban and rural environments [15]. Clinical manifestations of allergy, their development, severity and progression result from the interplay of multiple factors, including maternal–fetal interactions, individual genetic and epigenetic status and environmental and ambient influence related to climate change, as well as dietary habits reflected in the microbiome [16,17,18,19].

Diagnosis of allergy is based on history taking, physical examination and diagnostic tests. During the diagnostic process, the use of appropriate tools to confirm IgE-dependent reactivity is necessary. Moreover, IgE-mediated reactivity results should be interpreted in the context of exposure to the presumed culprit allergen. Despite the broadening arsenal of molecular diagnostic tests, skin prick tests, which have a long history of utility in allergy practice, remain the core diagnostic tool in allergology, helpful in identifying environmental factors triggering allergy symptoms.

The use of standardized allergen extracts ensures reproducible results, as well as good specificity, sensitivity and low cost of testing. They also feature high safety with a low risk of systemic reactions. The results of skin tests usually show good correlation with the results of nasal, conjunctival, cutaneous, oral and bronchial provocations; however, in some cases discrepancies between symptoms’ history and seasonal presentation and the results of specific IgE measurement may pose a diagnostic challenge, as observed during allergic IgE-mediated reaction limited to local mucosa without systemic involvement [7,20]. SPTs (together with serum-specific IgE and basophil activation tests) may also be considered one of the tools helpful in predicting the clinical reaction upon exposure to allergen, thus contributing to increased safety and efficacy of allergy diagnosis [21,22,23,24]. In addition, complete allergy diagnosis with the use of SPTs and specific IgE to allergen components can be valuable in predicting response to allergen immunotherapy as well as in assessment of the clinical significance of given allergen in eliciting clinical symptoms of allergy [25,26,27,28,29,30,31].

## 2. Materials and Methods

### 2.1. Study Design and Location

This study is a retrospective analysis of the frequency and distribution of IgE-dependent reactivity to allergens included into a standard panel of inhalant allergen extracts used for initial diagnostic assessment of patients referred to a tertiary outpatient allergy center in Poland. The center is affiliated with the University Hospital in the third largest city of Poland in terms of number of inhabitants located in the center of the country. The city area is 293.3 km^2^ and the population, as of 30 June 2024, consisted of 645,700 inhabitants [32]. Data were further analyzed in relation to sex, age and final diagnoses, as established by an allergist and recorded in accordance with the ICD-10 classification [33]. The medical diagnoses considered in analyses included allergic rhinitis conjunctivitis, asthma, atopic dermatitis, chronic rhinitis, rhinopharyngitis, chronic sinusitis, nasal polyps, cough, urticaria, anaphylaxis and other unspecified. Data from patients with suspected allergies referred by primary physicians were screened for suitability. The analysis used data from the archived medical records of 1711 patients diagnosed and treated in the four-year period between 2017 and 2020. Patients attending the outpatient clinic were recruited, provided their respective medical records met the eligibility criteria, as listed below. No other specific selection criteria were employed. Criteria of data eligibility included detailed demographic description, complete description of symptoms’ history and results of skin prick testing with airborne allergens panel (see below). Patients whose medical records were analyzed were permanent residents of the city of Łódź or its urban agglomeration.

### 2.2. Skin Prick Tests

The IgE-reactivity profile was determines by skin prick tests using an allergen kit from Allergopharma (Reinbek bei Hamburg, Germany). The test panel included the following eleven extracts of inhalant allergens: house dust mites *Dermatophagoides pteronyssinus* and *Dermatophagoides farinae*, alder (*Alnus glutinosa*), hazel (*Corylus avellana*), birch (*Betula verrucosa*), grasses mix (*Poaceae*), rye (*Secale cereale*), mugwort (*Artemisia vulgaris*), cat (*Felis domesticus*), dog (*Canis familiaris*) and *Alternaria alternata* mold spores, along with positive (histamine) and negative (diluent) controls.

Selection of the eleven allergen extracts included in the study was mainly driven by local aerobiology, previous studies and availability of extracts. This panel of allergens is commonly used—with small variations—in most allergy centers in our country as the first-line diagnostic tool in the assessment of patients with a suspected respiratory allergy.

The SPT procedure was performed in accordance with the established guidelines [34]. Histamine dihydrochloride (10 mg/mL) was used as the positive control and the solvent of the extracts was the negative control. Wheal size of 3 mm or greater than the negative control was considered positive, as it is commonly accepted in allergy diagnosis recommendations [35,36].

### 2.3. Statistical Methods

Allergic sensitization profiles were assessed in all subjects and further analyzed in subgroups created basing on sex and age. Wheal diameters in SPTs with each allergen were considered initial explained variables. They were subsequently reduced to four independent principal components using factor analysis.

These served as explanatory variables for testing within the gender and age of the participants. Two-way analysis of variance was used for this purpose.

Generalized methods (binomial distribution with a linking logit function) were used to assess the differences between proportions in the prevalence of allergic rhinitis and allergic asthma by gender and age categories of subjects.

Wherever there were more than two comparisons, an appropriate correction was applied (e.g., Benjamini–Hochberg corrects). However, all comparisons concerning proportions (chi-square and generalized linear/non-linear methods) in the binomial distribution are equivalent to testing proportions, so there is no need to test odds ratios (ORs). Odds ratios (ORs)—where applicable—were estimated with confidence intervals.

Factor analysis was used to reduce the number of variables. Principal components were selected as factors. Eleven dependent variables (positive SPTs) were reduced to four independent factors: PC1, PC2, PC3 and PC4. These factors replaced the variables with which they are most strongly related through factor loadings. Factors were named according to factor loading values ≥ 0.7. This method of variable reduction does not result in a loss of explained variability. Another advantage of principal components is that they are independent of each other, which cannot be said for the original variables.

Statistical significance was assumed at the level of α = 0.05 and the confidence interval at the level of 0.95. In the case of more than two categories, the Benjamini–Hochberg post hoc test was performed using PQStat Software 2023 v1.8.6 (PQStat Software, Poznań, Poland). All other calculations were performed using Statistica 13.3 (StatSoft Polska, Kraków, Poland).

Chi-square tests were then performed between the disease entities determined by the allergists and the SPT results. Additionally, odds ratios with confidence intervals were calculated.

## 3. Results

### 3.1. Distribution of Sex and Age Groups

**Figure 1:**
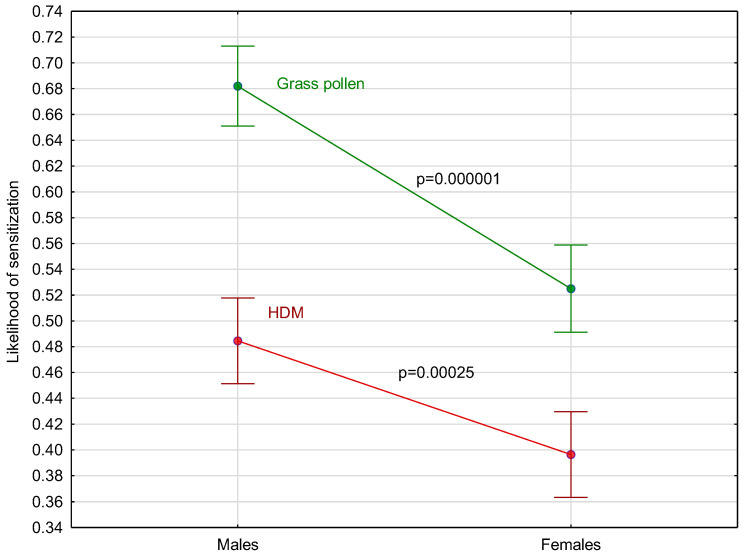
Sex differences in likelihood ratio of grass pollen reactivity and house dust mites reactivity in the entire study group. HDM, house dust mites.

The studied population was characterized with the following sex and age distribution. Females accounted for 49% (n = 840) and males for 51% (n = 871) of patents. The mean age in the entire study group was 30.25 ± 6.95 years (age range: 2.57–86.42). The mean ages of males and females in the analyzed population were 27.90 ± 6.35 (age range: min.: 2.92; max.: 86.42) and 32.69 ± 17.2 (age range: min.: 2.57; max.: 77.97) years, respectively. Males were significantly more likely to be sensitized to grass pollen and house dust mites (Figure 1).

For further analysis, subjects were subdivided into three age subgroups, as presented in Table 1. The age distribution shows a predominance of patients aged 10 through 50 years, who constituted 74.81% of the group, followed by the age group 50 years and older, accounting for 14.1% of the studied population. Further details regarding the study group are provided in Table 1.

### 3.2. Reactivity Profiles in the Entire Study Group

**Figure 2:**
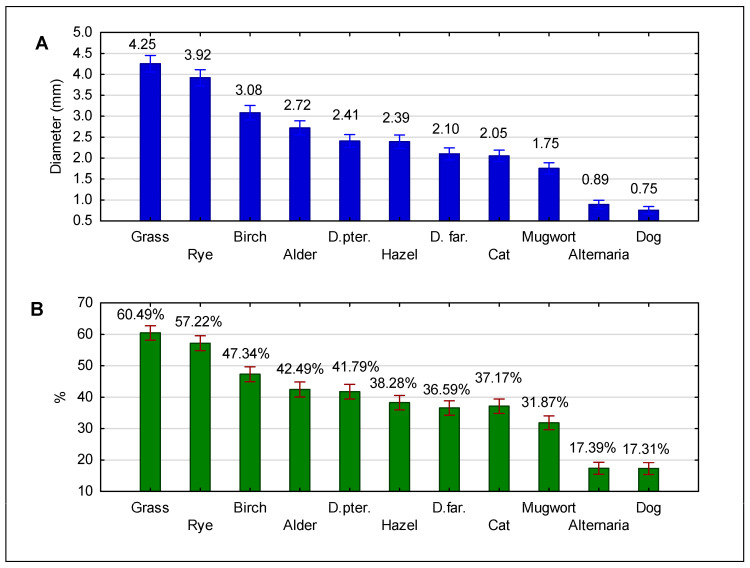
Panel (**A**) Diameters of wheal in skin prick tests with airborne allergens from the standard panel. Mean values (in mm) are shown as numerical values; 95% confidence intervals are indicated with whiskers. Panel (**B**) Frequency of positive skin prick test results with airborne allergens from the standard panel in the entire study group (n = 1711). D. pter., Dermatophagoides pteronyssinus; D. far., Dermatophagoides farinae.

The highest IgE-reactivity frequencies were observed in cases of allergens of grass, rye, birch and alder pollen, and of *Dermatophagoides pteronyssinus* mite. The lowest frequencies of reactivity were found for allergens of dog and *Alternaria*. A detailed summary of the frequency of reactivity profiles and average wheal diameter are presented in Figure 2.

**Figure 3:**
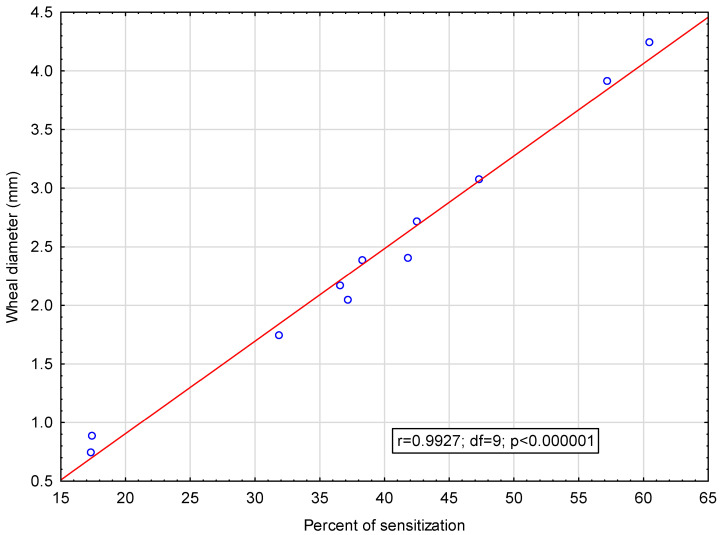
Results of the Pearson correlation between mean wheal diameter in skin prick tests with a given allergen in the entire study group (n = 1711) and percent of positive skin prick results with the same allergen (r = 0.9927, r^2^ = 0.9855, *p* < 0.000001).

Wheal diameters in SPTs were also higher with regard to allergens with which the SPT results were positive most frequently. In the entire study group, as shown in Figure 3, a significant positive correlation has been observed between the mean wheal diameter of positive SPTs with a given allergen extract and the percentage of positive SPT results with that allergen.

### 3.3. Monovalent and Polyvalent Reactivity

#### 3.3.1. Monovalent Reactivity Profiles

**Figure 4:**
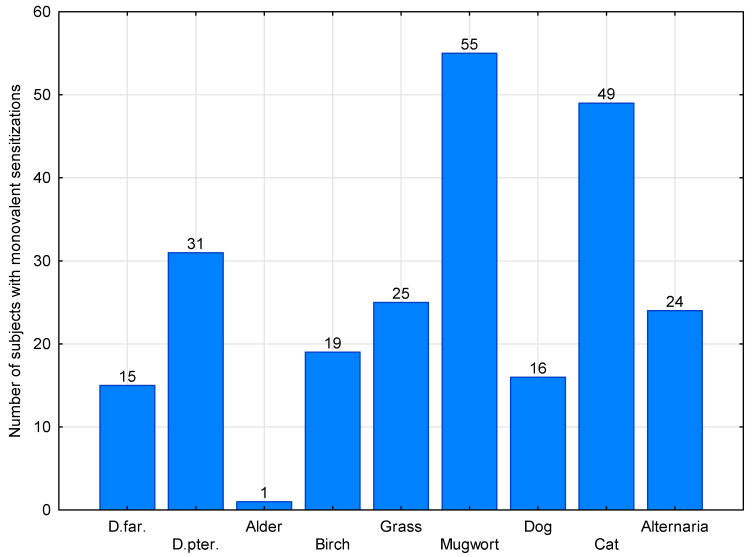
Distribution of the numbers of monovalent reactivity to airborne allergen extracts in the entire study group (n = 1711). No monovalent sensitization was observed in the case of rye pollen and hazel pollen. The percentage profiles is given in Section 3.3.1.

Monovalent reactivity was found in 13.73% of the participants. As shown in Figure 4, the most common monovalent IgE-reactivity reactions in the entire study group were found in the following order: to mugwort pollen: 3.26% (n = 55) followed by cat allergens: 2.9% (n = 49) *D. pteronyssinus* mites. 1.84% (n = 31), grass pollen 1,48%, *Alternaria* 1,42%, birch pollen 1.12%, dog 0.95%, *D. farinae* mites 0,89% and alder pollen 0,006%. The number of monovalent reactivity was highest in age group II (10–50 years) and differed significantly from the remaining age groups (χ^2^. = 52.5, df = 1, *p* = 0.000001, OR = 3.16 (2.29–4.36). Distribution of monovalent reactivity among age groups is presented in Table 2.

#### 3.3.2. Polyvalent Reactivity Profiles

**Figure 5:**
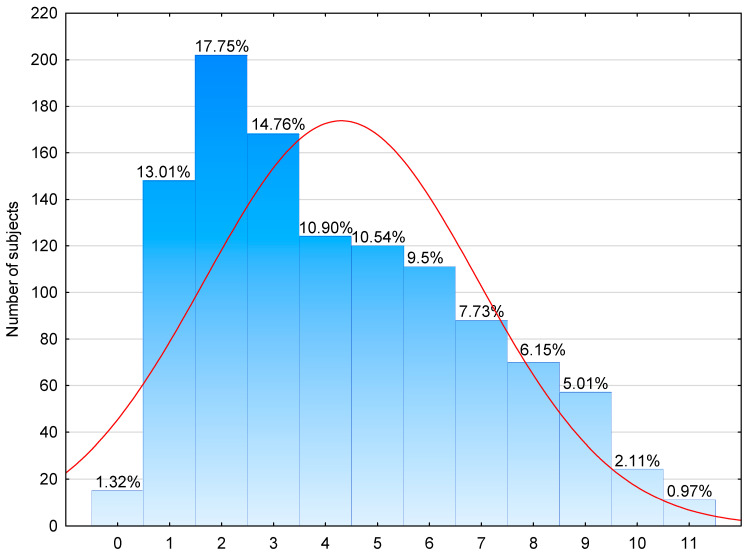
Distribution of the numbers of reactivity (lower X axis) in skin prick test panel in the entire group (n = 1711). Number of subjects with given number of reactivity is indicated on the Y axis with percentage of the total group indicated above each column.

As many as 98.7% of the studied population had one or more positive SPT results. Polyvalent reactivity to two or more allergen extracts was found in 86% of subjects. Most often, in 17.8% of subjects, two positive SPT results were ascertained. Reactivity to all eleven allergen extracts was found in 1% of patients (Figure 5).

### 3.4. Factor Analysis

Data reduction using factor analysis allowed for the extraction of four principal components (factors) described below, which were considered as explained variables.

- Factor I was associated with positive SPT results to tree pollen extracts (alder, hazel, birch).
- Factor II characterized allergy to mites (Dermatophagoides pteronyssinus and Dermatophagoides farinae).
- Factor III was associated with allergies to grass pollen extracts: mix and rye.
- Factor IV determined allergies to animal allergens: dog and cat.

Sex and age were considered as explanatory variables.

**Figure 6:**
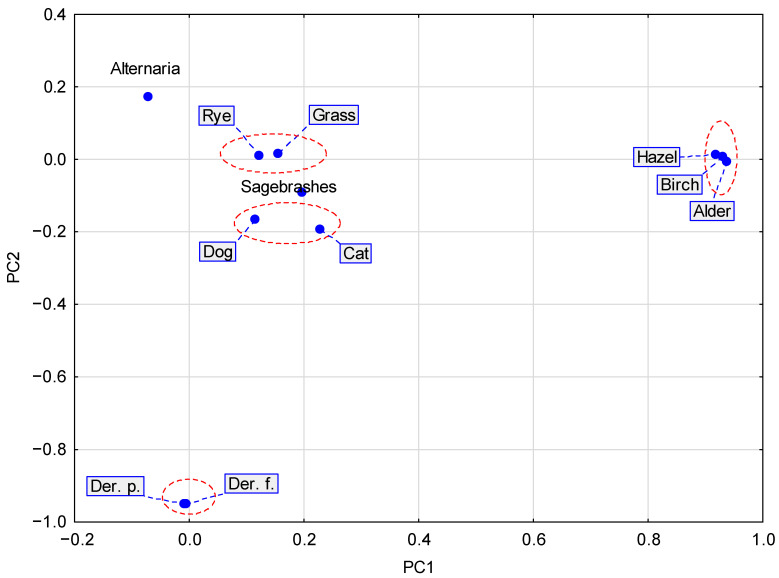
Graphical representation of the results from Table 3 for the first two factors PC1 and PC2, which explain the most variability. The charges marked with a dashed line characterize four distinct reactivity groups.

The four independent principal components identified in the factor analysis allowed us to conclude that the wheal diameters for grass/cereals and mites were higher in men (*p* < 0.0001). For mites, the largest wheal diameter was obtained in age group II (10–50 years). It was significantly different from age group I (<10 years (*p* = 0.0045) and age group III (>50 years) (*p* = 0.006). The results of the factor analysis are presented in Table 3 and Figure 6.

#### 3.4.1. Testing of the Four Extracted Factors in Relation to Age, Sex and the Extracted Age Categories

##### Factor I—Allergy to Tree Pollen

The highest wheal diameters to tree pollen extracts were found in the age group 50+. They were significantly higher than in the youngest patients (*p* = 0.0048) than in the members of the age group 10–50 years (*p* = 0.000003). No significant differences were found in relation to the sex of the subjects (Figure S1).

##### Factor II—Allergy to House Dust Mites

For factor II, i.e., mite extracts, the highest reactivity rates were found in 10–50-year-olds. Statistically significant differences were noted with the youngest group (*p* = 0.045) and the oldest group (*p* = 0.016) (Figure S2A). Significantly higher values of wheal diameters were also noted in males (Figure S2B).

##### Factor III—Allergy to Grass and Rye Pollen

In cases of grass and rye pollen allergens, the highest value of wheal diameter was observed in age group II. Statistically significant differences occurred between all age categories: between category I and II (*p* = 0.0004); between II and III (*p* = 0.00002), and between I and III (*p* = 0.0026). Positive reactivity was significantly more frequent in men (*p* > 0000001) (Figure S3A,B).

##### Factor IV—Allergy to Pet Allergens

In cases of reactivity to pet allergens (dog/cat), the highest frequency was found for age category II (10–50-year-olds). Significant differences were obtained between age groups I and II, as well as II and III: *p* = 0.023 and *p* = 0.019, respectively. No significant differences were noted between the sexes (Figure S4).

### 3.5. Frequency of Clinical Diagnoses According to the ICD-10 Classification

From the archival patient charts, information was obtained that all patients included in the study were diagnosed with atopic diseases of atopic origin. These were allergic rhinitis, conjunctivitis, bronchial asthma and atopic dermatitis. Some patients were diagnosed with chronic rhinopharyngitis, chronic sinusitis, nasal polyps, cough, urticaria and other unspecified. The association of disease entities with the studied reactivity to specific inhalant allergens was estimated using the odds ratio (OR ± 95% CI). Detailed results are presented in Table S1.

### 3.6. Clinical Diagnoses in Relation to SPT Results

#### 3.6.1. Allergic Rhinitis (AR)

In the study group, the chance of developing AR (J30) was more than three times higher in the case of grass allergy [OR = 3.24 (2.58–4.05), *p* < 0.000001] and more than two times with regard to rye, hazel and alder pollen allergens than in subjects without such reactivity.

It was found that the risk of allergic rhinitis had a significant relationship with reactivity to all extracts of inhalant allergens. It was most strongly associated with grass and rye pollen allergy, respectively, OR = 3.24 (2.58–4.05), *p* = 0.000001 and OR = 2.92 (2.33–3.65), *p* = 0.000001, followed by hazel pollen, OR = 2.26 (1.77–2.82), *p* = 0.000001, and alder, OR = 2.22 (1.75–2.82), *p* = 0.000001, as well as *Alternaria*, birch, cat and mites (Table S1). Regarding sex and age groups, AR diagnosis was significantly associated with male sex (Figure S5) and age group >50 years, as compared with the other age groups (Figure S6).

#### 3.6.2. Asthma

The risk of developing asthma was associated to the highest extent with reactivity to allergens coming from dog (OR = 2.28 (1.75–2.98), *p* < 0.000001), cat (OR = 1.96 (1.60–2.41), *p* < 0.000001) and house dust mites (OR = 1.49 (1.21–1.82)). Regarding sex and age groups, asthma was significantly associated with female sex (Figure S5) and age groups 10–50 years and >50 years, as compared with the youngest group (Figure S6).

#### 3.6.3. Other Atopic Conditions

The risk of the diagnosis of allergic conjunctivitis was associated with reactivity to hazel [OR = 1.86 (1.29–2.70); 0.0025], alder [OR = 1.86 (1.29–2.70)] and birch [OR = 1.54 (1.07–2.24)]. Regarding diagnosis of atopic dermatitis, its risk was highest in subject allergic to birch [(OR = 2.12 (1.36–3.25), *p* < 0.001) and alder (OR = 1.70 (1.11–2.60), *p* = 0.014)]. Negative associations between reactivity to different allergens and the risk of being diagnosed with a given condition were found with regard to chronic rhinopharyngitis (ICD-10: J31), chronic sinusitis (J32), nasal polyps (J33) and cough (R05). For example, in people with nasal polyps, the chance of being allergic to grasses was estimated to be four times lower than in people without polyps (Table S1). For ICD-10 codes, L50 (urticaria) and T78 (adverse effects, not elsewhere classified—which includes among others, anaphylactic shock, food hypersensitivity, different forms of angioedema and unspecified allergy), no significant associations were ascertained.

## 4. Discussion

In our retrospective study, we considered the results of skin prick tests and physicians’ diagnoses in 1711 patients referred by primary physicians due to suspected immediate-type allergy. It should be emphasized and taken into account that these data come from a tertiary clinic and, as such, may overrepresent more severe allergy cases compared to the general population. In this analysis, we looked at the SPT results from different perspectives. Our cross-sectional studies, based on medical records, indicate that using SPTs is a valuable first-line approach in the assessment of subjects with suspected allergy to airborne allergens. According to Szmyd et al. [37], there are strong positive correlations between positive SPT results and serum-specific IgE concentrations, which allows for the diagnosis of inhalant allergy in 95% of cases. In addition, SPT results show good correlation with the results of nasal, conjunctival and other provocation tests [38].

In our group, the high prevalence of reactivity to pollen extracts, mainly grasses and rye, is quite surprising. In previous studies from our city and province, the highest frequency of reactivity was seen with regard to house dust mites, both in urban and rural pediatric populations. Similar results were also obtained by other researchers from our country. The highest prevalence of dust mite reactivity was found in studies of children and adults from the city of Krakow and among adult patients from the city of Bydgoszcz and the surrounding area [39,40]. Additionally, in patients with AD, the most common positive SPT result was observed during testing with *D. pteronyssinus* and *D. farinae*, while reactivity to grasses was the third most frequently ascertained [41]. It is suspected that such discrepancies result from differences in the recruitment of the studied groups. Diagnostic screening studies and epidemiological studies show, however, a high degree of repeatability [42].

The allergic reactivity profiles observed in this study indicate the greatest significance of grass pollen allergens in causing IgE-reactivity in the population of central Poland. These results are consistent with the results of the nationwide ECAP study conducted in the general population, where grass pollen allergens were shown to be the clinically most important group of aeroallergens in Poland. In the ECAP study, it was found that 21% of participants had positive SPTs to grass extracts, and 16% of them reported suggestive symptoms during the pollen season [43]. The *Poaceae* (grass) pollen seems to increase its expansiveness and is currently considered to be the leading biological pollutant in atmospheric air and the main cause of pollen allergy in Europe and the world [44,45]. The grass allergens described so far (13 groups) have been divided into water-insoluble ones, which seem to play a role in the centrally mediated inflammatory response, and water-soluble allergens, which are probably involved in the peripheral humoral response [46,47]. Pollen NADPH oxidase (which induces pollen tube growth) has also been shown to have a pro-inflammatory effect, independent of adaptive immune responses [48]. Animal studies have shown that activation of pollen NADPH affects the generation of reactive oxygen species and played an important role in the pathogenesis of allergy [49]. Additionally, air pollution and other biotic (e.g., microorganisms and viruses) and abiotic factors may influence pollen allergenicity. For example, they may contribute to oxidative stress, both to pollen and to human epithelial cells, which may promote allergy to grass and other plant pollen [50]. It has also been shown that pollen lipids can be internalized by dendritic cells presented by CD1 to NKT cells, which release IL-4 and promote the differentiation of Th0 lymphocytes into Th2 cells [51].

In addition, the allergen extracts used during SPTs contain proteins that have not yet been characterized, and so far we do not have the ability to assess their reactivity with IgE from patient sera. For example, recent proteomic analyses indicate the presence of up to 3000 newly identified proteins for each pollen taxon, which may contribute to their different allergenic potential [52].

### 4.1. Monovalent Reactivity

It is interesting that the most common monovalent reactivity was found to mugwort extract (*Artemisia*), with a general frequency of reactivity of 3.2% in the entire study group. Mugwort is a common plant in Poland, blooming from July to September, together with grasses and other weeds [53,54,55,56], which complicates management of allergy with reactivity to this taxon. The participation of mugwort pollen in the aeroplankton of the atmospheric air in our city has never been high during more than 20 years of pollen monitoring. Its influence on the development of IgE-reactivity has not been analyzed in more detail either. However, reports from Asian countries indicate that mugwort pollen allergens are considered clinically significant in the context of allergic disease development. For example, the frequency of reactivity to mugwort in northern China was estimated at 26–48% [57,58]. So far, seven potential mugwort pollen allergens with IgE binding ability have been identified [59]. The high cross-reactivity of mugwort allergens is also known. Available studies show that some allergen components of mugwort and ragweed may be mutually cross-reactive (e.g., Art v 1 with Amb a 4 and Art v 4 with Amb a 8) [60,61]. Aerobiological studies conducted in our city have indicated generally low exposure to ragweed pollen, which comes from long-distance transport, mainly from southern and eastern Europe, and showed that the pollen seasons for mugwort and ragweed are separated [55,62]. It may, therefore, be speculated that, in our region, reactivity to the Art v 1 mugwort pollen component can be a proverbial Trojan horse that would promote reactivity to the Amb a 4 ragweed pollen component.

In our population, we have identified a notable difference between the amount of monoreactivity to *D. farinae* and *D. pteronyssinus* mites. Considering the fact that allergens coming from these mites are highly cross-reactive, this difference may seem unexpected and discrepant. We can, however, speculate that it is associated with the influence of external factors (e.g., housing conditions and some biotic and abiotic indoor factors, including humidity, temperature and presence of microscopic fungi) upon the composition of the local acarofauna [63]. Our unpublished research shows that *D. pteronyssinus* is represented to a higher extent in the house dust samples collected in our city’s apartments, compared to *D. farinae.* Other published data [64] show that in Poland, generally, older buildings and furnaces are more conducive to the presence of *D. pteronyssinus*, while new buildings with central heating systems are more favorable milieu for *D. farinae*. Nevertheless, in our city *D. pteronyssinus* mites were the dominant taxon. One may, therefore, assume that the rate of specific IgE-reactivity to either house dust mite can be associated with the level of exposure and reflect features of local acarofauna resulting from housing conditions and external modifying factors, as mentioned above.

Employing only skin prick testing to allergen extracts, and not the assessment of reactivity to allergen components, may be considered a limitation of the study. At the time when the patients had been assessed, component-resolved diagnostics was much less common and accessible in routine diagnostic management; therefore, the number of performed assessments of the levels of IgE specific to allergen components was not considerable and could not be subject to extensive analysis. Basing the assessment of mono- and polyreactivity solely on SPT positivity leaves out possible cross-reactivity between allergenic components belonging to the same groups and represented in different allergen sources, such as trees and furry animals. (e.g., PR-10, profilins, lipocalins). In this context, it should be taken into account that IgE-reactivity to alder and hazel allergen extracts may be in large part due to cross-reactivity of their allergens with birch allergens, whereas the majority of subjects apparently allergic to hazel and/or alder are, in fact, primarily reactive to birch pollen.

Cross-reactivity as a plausible explanation of apparent polysensitization to tree allergens is further supported by significant gamma-correlations between the compared frequencies of positive SPTs to alder and hazel, alder and birch and birch and hazel pollen (*p* < 0.00001). However, with regard to mugwort pollen, a very weak correlation was found between the number of positive SPTs with mugwort and the number of positive SPT results with all of the trees present in the allergens panel. Results of the additional gamma-correlation analysis between positive SPTs with extracts of potentially cross-reactive allergen sources are presented in the Supplementary Material as Table S2.

This issue requires a broadened and deepened separate analysis. Considering this, patient diagnostics should be enriched with an assessment of clinical significance, molecular studies and determination of possible cross-reactions.

### 4.2. Reactivity and Age

The division into three age groups made in this study showed heterogeneity in the allergy profiles. Surprisingly, the identification of three age clusters showed that in subjects aged over 50, the highest frequencies of allergies to grass and rye were observed. This is difficult to explain using known immunological mechanisms. Several studies show that in senior age groups a progressive loss of the ability to respond to antigen stimulation as well as immune dysfunction are observed [65,66].

### 4.3. Atopy and Sex

In our study, the frequency of one or more positive SPT responses was higher among males compared to females in each decade of life. This contrast suggests that factors other than type I hypersensitivity reactions are responsible for the differences. Numerous scientific studies have analyzed multidimensional immunological and inflammatory mechanisms. Sex differences can manifest themselves at different levels, mainly genetic, anatomical and endocrine. Many researchers emphasize that sex hormones, such as estrogen and testosterone, can affect respiratory diseases [67,68], but their effects are multifactorial, e.g., dependent on age, menopause, obesity, stage of the menstrual cycle, etc. It is known that sex hormones mediate cellular and humoral immune pathways and may partially explain the increased frequency of inflammation in women compared to men. Studies indicate that estrogen may contribute to airway inflammation in women with asthma, while testosterone may have a protective effect in men [69].

In the National Health and Nutrition Examination Survey (NHANES), serum testosterone levels were found to be inversely associated with the risk of developing current asthma in both sexes [70]. In males, it was reported that each 1-unit increase in testosterone levels reduced the odds of developing asthma by 11%. In general, testosterone in men may reduce Th2 airway inflammation by reducing IL-13 expression and eosinophil infiltration in the airways [71].

### 4.4. SPT Results and Allergic Diseases

It should be emphasized that reactivity to hazel, alder and birch pollen is positively associated with conjunctivitis. In the context of newer studies, the eye cannot be neglected or omitted in the context of allergic diseases and conjunctival symptoms, as a complication of allergic rhinitis should be given more recognition. It was found that proteases from hazel and birch pollen, as well as the grains of other taxa, can induce both IgE-dependent and IgE-independent reactions [72]. They can contribute to the decomposition of tear fluid proteins and damage to the epithelial cells, in particular in polluted urban environments [73].

It is believed that the development of allergies may be influenced by various exogenous—mainly environmental—factors, e.g., progressive urbanization, lifestyle changes, increased exposure to air pollution and allergens [74]. Our previous studies show that exposure to pollen seems to be of particular importance in already sensitized subjects. However, it must be underlined that clinical symptoms mainly occur when the level of exposure exceeds the individual threshold level. Additional factors may also contribute to the development of allergy symptoms. For example, Olivieri et al. [75] showed interactions between atopy, seasonal exposure to pollen, and FeNO, total IgE and FEV1/FVC levels, which were of a dose-response nature.

Our studies show that the risk of allergic rhinitis, conjunctivitis and atopic eczema was strongly associated with reactivity to pollen of locally flowering plants. Although seasonal exposure to birch pollen in our region is almost twice as high, but shorter in duration than grass pollen (unpublished data), grass and rye allergens turned out to be the most aggressive, more often promoting allergy symptoms in our group.

In parallel to diagnostic and epidemiological studies, our center conducts aerobiological studies in the city center. The main grass pollen season in Poland lasts about 80–90 days between the end of May, throughout June until mid-July. Data from the years 2003–2024 indicate a significant increase in grass pollen concentrations over a more than twenty-year period (*p* = 0.045; r^2^ = 0.186; unpublished data), while birch pollen concentrations show two-year fluctuations, but without a significant increase in concentrations over the last 22 seasons.

A recent study by Klangkalya et al. [76] indicated that, in children, only the cut-off value of the wheal in SPTs of 6.6 mm provided 100% specificity for predicting AR induced by HDM. However, in our work, the wheal size criterion of the positive SPT result was assumed at ≥3 mm. Perhaps in future studies different values may be used as criteria for analyses.

It should again be emphasized that the risk of developing allergies depends on many factors, including genetic, epigenetic, prenatal, perinatal and environmental ones, and their mutual interactions [15,77]. Growing up in a polluted urban environment confers an increased risk of developing allergies in early childhood. In our previous studies, differences in the frequency of allergies in children were found depending on the region of residence within the same city. They were significantly higher in the downtown district compared to districts located far from the city center [78]. Similar findings, i.e., differences in pediatric asthma incidence between districts of the same town differing in terms of proximity to industrial facilities, have recently been described in another region of Poland [79].

In addition, the results of the study by Stelmach et al. [80], conducted in our city among children living in an orphanage, indicated a low frequency of atopy (12.5% positive SPT) and atopic diseases. For example, seasonal allergic rhinitis was found in only 2.5% (3 out of 120) of children. Similarly, in children living in foster care homes the presence of atopic diseases was observed in 11.3%, which is almost half as many as in the control population, in children living with their parents (26%), despite the fact that all children came from the same area of the city. The authors suggest that exceptionally difficult living conditions and multiple infections in early childhood may lead to lower reactivity in later life [81].

In our study, we have not addressed or interpreted the data on symptoms occurring upon exposure to allergens. Similarly, the severity of symptoms of allergic disease has not been interpreted in the exposure or SPT result context. This study focused on the association between IgE-reactivity and the presence of diagnosis of given condition, as per available medical records. Such an approach, i.e., based on the presence of given diagnosis in medical records, always bears the possibility of bias or error. However, retrospective data from large populations are likely to be recorded in a non-standardized manner, unless a uniform scheme of their collection is established beforehand. This was not the case here, which should be considered as a factor influencing further clinical interpretation of these data.

## 5. Conclusions

Our study is representative of the urban population of central Poland and offers a platform for the assessment of the relationships between IgE-reactivity and allergic disease. Identification of sensitizing allergens, i.e., exposure risk factors for the development of symptoms, is crucial for allergy diagnostics. Reactivity profiles determined using a basic panel of skin tests may vary depending on sex, age groups and individual disease entities of allergic origin. The results of our study can serve as an objective reference to the frequency of allergen occurrence in the urban environment in central Poland, but also in similar climate and geographical conditions. Accurate diagnosis combined with optimal therapy requires the use of appropriate tests confirming allergen reactivity and obtaining detailed information on exposure to the presumed culprit allergen.

The results of this study can, therefore, be summarized in the following main points:

- According to the results of SPTs, the most important sensitizing allergens in allergic subjects in central Poland were the pollen from grass (60.5%), rye (57.22%) and birch (47.34%).
- The most common monovalent reactivity found was that to mugwort pollen.
- The frequency of one or more positive SPT results was higher among males.
- The age distribution shows the highest reactivity of allergies in the age group 10–50 years, with male predominance
- In patients with clinically diagnosed AR, the probability of developing symptoms was higher in those sensitized to grass pollen, rye, alder, hazel, *Alternaria*, birch, cat, *D. pteronyssinus* and *D. farinae* allergens.
- Higher odds of developing asthma were associated with positive SPT results with dog, cat and dust mite allergens.
- Higher odds of developing atopic dermatitis were associated with positive SPT results with birch and alder pollen allergens.
- Higher odds of developing allergic conjunctivitis were associated with positive SPT results with alder, hazel and birch pollen allergens.

Due to the high prevalence of allergic diseases in Poland and worldwide, there is an urgent need to study the spectra of reactivity and their relationship with allergy. Identification of the most frequent reactivity profiles associated with target-organ manifestations of allergic disease in a given geographical area may provide valuable descriptive data with regard to a description of clinical phenotypes of allergy. Trends in the prevalence of reactivity to specific allergens depending on age and physician-diagnosed allergic disease in Poland have rarely been studied in both children and adults. Our research focuses on the reactivity profiles in individual allergic diseases, as well as age- and sex-related differences in patients diagnosed and treated in an outpatient allergy clinic. In this context, it adds to the current state of knowledge on the epidemiology and distribution of reactivity to the most common airborne allergens in Central Europe. Further studies in this geographical area are needed in order to cover a broader spectrum of reactivity and to analyze possible variations and fluctuations in allergen reactivity profiles over longer periods. This aspect of studies on allergen reactivity profiles deserves particular recognition in the light of climate changes, which may affect the extent and patterns of exposure to seasonal airborne allergens. We can foresee that climate change-associated shifts in the IgE-reactivity profile extended over longer periods may incur modifications in recommended immunotherapy schemes and enforce changes in routine diagnostic work-up with regard to allergen reactivity assessed through SPT and serum-specific IgE concentrations.

## Figures and Tables

**Table 1:** Basic characteristics of the entire study group, as per division into age groups. SD, standard deviation.

	Group I <10 years	Group II ≥10–50 years	Group III >50 years
Number of subjects (n)	190	1280	241
Mean age ± SD	6.87 ± 2.0	28.11 ± 11.06	60.02 ± 6.65
Age range (years)	2.57–9.99	10.04–49.96	50.28–86.42
% of the total population	11.1%	74.81%	14.09%

**Table 2:** Monovalent reactivity as per division into age groups.

	Number of Subjects with Monovalent Reactivity in a Given Group	Percentage of Subjects with Monovalent Reactivity in a Given Group
Group I <10 years (n = 190)	26	13.68%
Group II ≥10–50 years (n = 1280)	140	10.94%
Group III >50 years (n = 241)	69	28.63%
Total (n = 1711)	235	13.73%

**Table 3:** Results of the factor analysis of SPT positive results with regard to four principal components (as described in Section 2.3). SPT, skin prick test; PC, principal component.

Variables		Factor Loadings Extraction Principal Components (PC). Marked Loadings > 0.7			
		PC1_Trees Pollen 1	PC2_ House Dust Mites 2	PC3_Grass Pollen 3	PC4_Pets 4
1.	*D. farinae*	−0.010730	**0.948330**	0.014072	0.080939
2.	*D. pteronyssinus*	−0.013067	**0.949340**	0.044677	0.050046
3.	Alder	**0.935816**	0.004489	0.190270	0.099719
4.	Hazel	**0.918262**	−0.010066	0.201667	0.092477
5.	Birch	**0.927786**	−0.014525	0.153930	0.102391
6.	Timothy	0.142750	−0.020950	**0.929728**	0.084068
7.	Rye	0.114222	−0.013704	**0.935631**	0.101022
8.	Mugwort	0.192302	0.069246	0.371463	0.048909
9.	Dog	0.142326	0.170201	0.021874	**0.715538**
10.	Cat	0.235508	0.186921	0.080663	**0.712500**
11.	*Alternaria*	−0.087785	−0.185442	0.122141	0.588676
	Explained variance	2.733860	1.904627	2.002449	1.423891
	Proportion of variance	0.248533	0.173148	0.182041	0.129445

## Data Availability

The data presented in this study are available upon reasonable request from the corresponding author (B.M.-W.).

## Supplementary Materials

The following supporting information can be downloaded at: https://www.mdpi.com/article/10.3390/medicina61091676/s1, Figure S1. Comparison of variance in mean wheal size between age categories with regard to tree (birch, hazel, alder) pollen allergens sensitization (Factor I). * *p* = 0.0048; ** *p* = 0.00003. Figure S2. Comparison of variance in mean wheal size in SPTs with house dust mites (*D. pteronyssinus*, *D. farinae*) allergens between age groups (panel A) and sexes (panel B); * *p* = 0.045; ** *p* = 0.016; *** *p* = 0.00008. Figure S3. Comparison of variance in mean wheal size in SPT with grass pollen allergens between age groups (panel A) and sexes (panel B); * *p* = 0.026; ** *p* = 0.0004; *** *p* = 0.00002; **** *p* = 0.00000005. Figure S4. Comparison of variance in mean wheal size in SPTs with cat and dog allergens (Factor IV) between age groups. * *p* = 0.023; ** *p* = 0.019. Figure S5: Differences between proportions in the prevalence of allergic rhinitis and asthma by sex categories of subjects. Figure S6. Differences between proportions in the prevalence of allergic rhinitis and asthma by age categories of subjects. Table S1. Associations between physician-diagnosed allergic/atopic conditions (as retrieved from archival patients’ charts) and allergic sensitization ascertained by skin prick tests (SPTs). Table S2. Gamma correlations for compared frequencies of positive SPT results (positive result defined as a wheal ≥3 mm diameter).

## Abbreviations

The following abbreviations are used in this manuscript:

SPT	Skin prick test
AR	Allergic rhinitis
ICD-10	International Statistical Classification of Diseases and Related Health Problems, Tenth Revision
NADPH	Nicotinamide Adenine Dinucleotide Phosphate
OR	Odds ratio
CI	Confidence interval
IL	Interleukin
HDM	House dust mite(s)
FeNO	Fractional exhaled nitric oxide
FEV1	Forced expiratory volume in one second
FVC	Forced vital capacity
