# Supplementary material for: Aeroallergen IgE-Reactivity Patterns and Respiratory Allergy in Children and Adults: A Retrospective Study in 1711 Patients from the Central Poland Urban Area
Source: Medicina (Kaunas). 2025 Sep 15;61(9):1676. doi: 10.3390/medicina61091676 (PMC12471568; doi:10.3390/medicina61091676)
Supplement: Supplementary file 1 [file medicina-61-01676-s001.zip › medicina-3788283-supplementary.pdf]

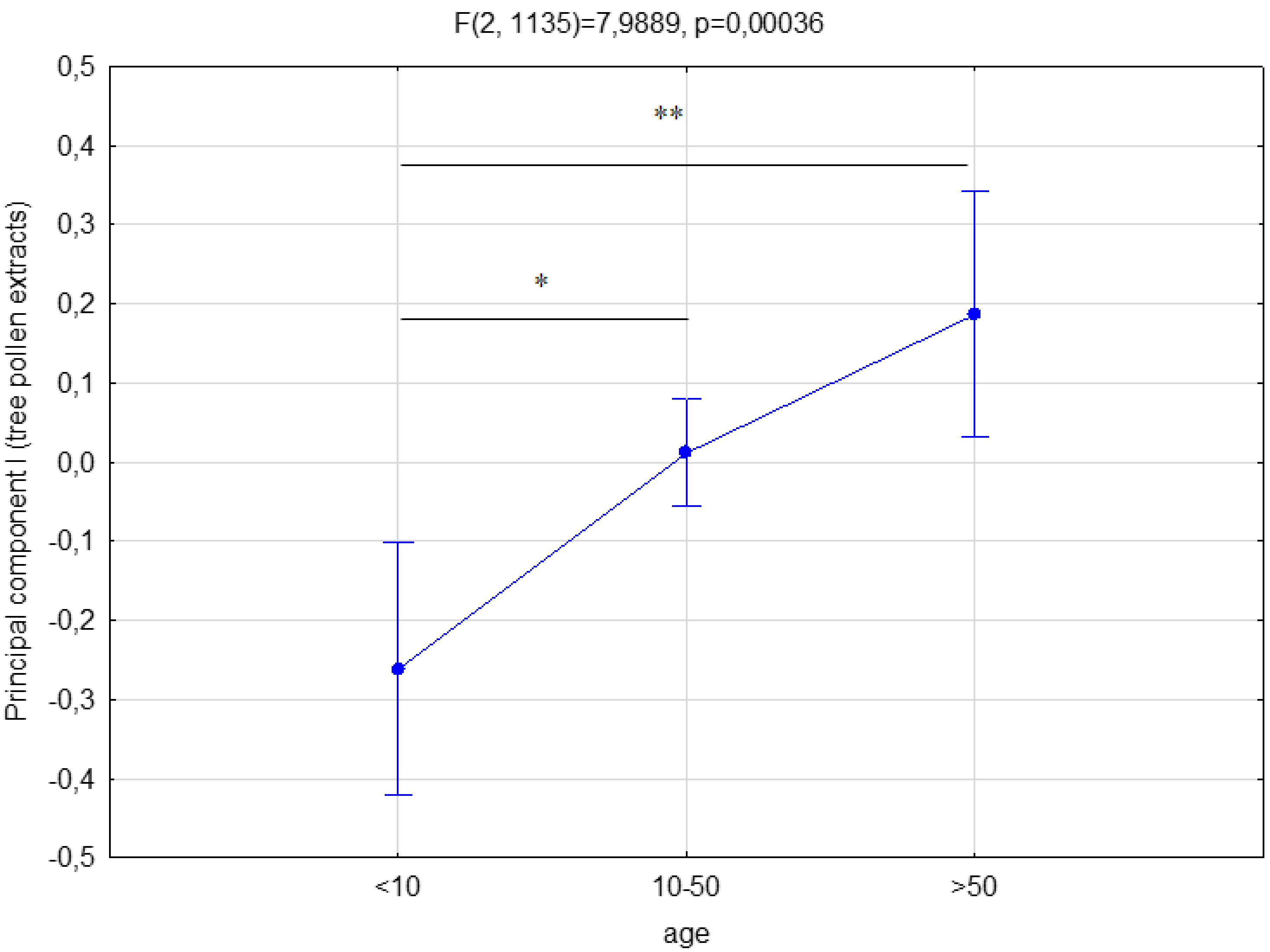

Figure S1. Comparison of variance in mean wheal size between age categories with regard to tree (birch, hazel, alder) pollen allergens sensitisation (Factor I**).** * p=0.0048; ** p=0.00003.

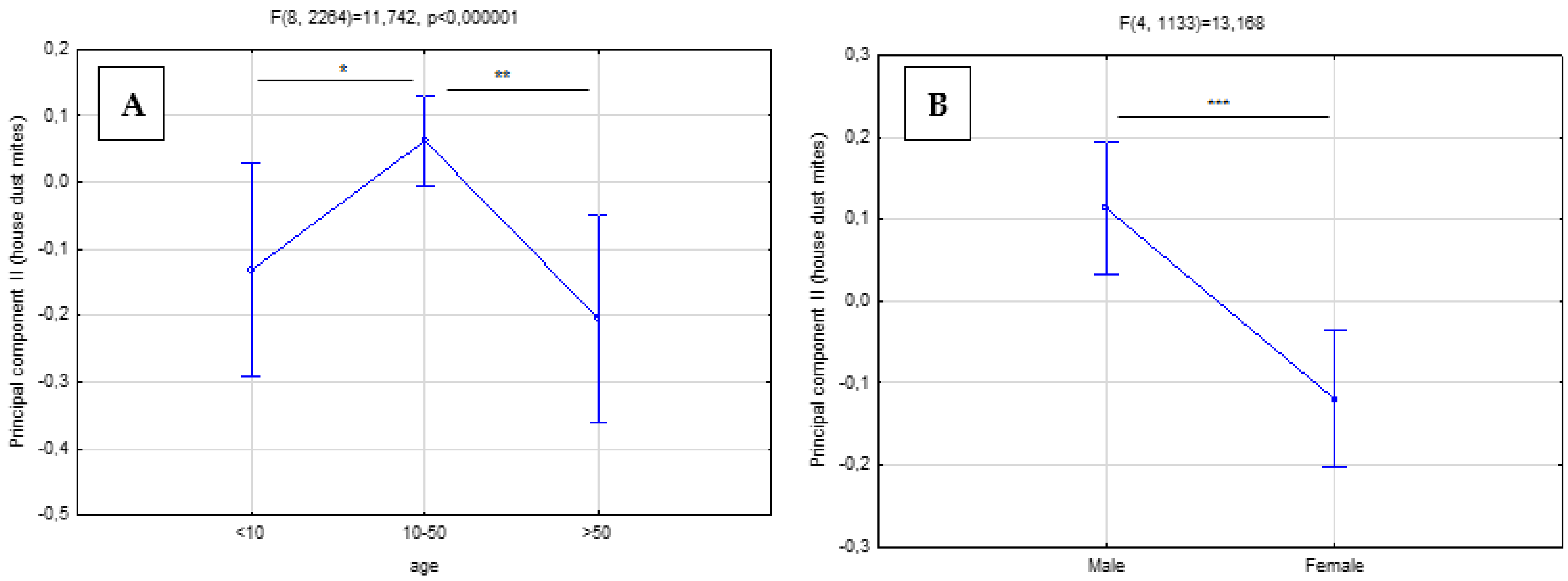

Figure S2. Comparison of variance in mean wheal size in SPT with hosue dust mites *(D. pteronyssinus, D. farinae)* allergens between age groups (panel A) and sexes (panel B); * p=0.045; ** p=0.016; *** p=0.00008

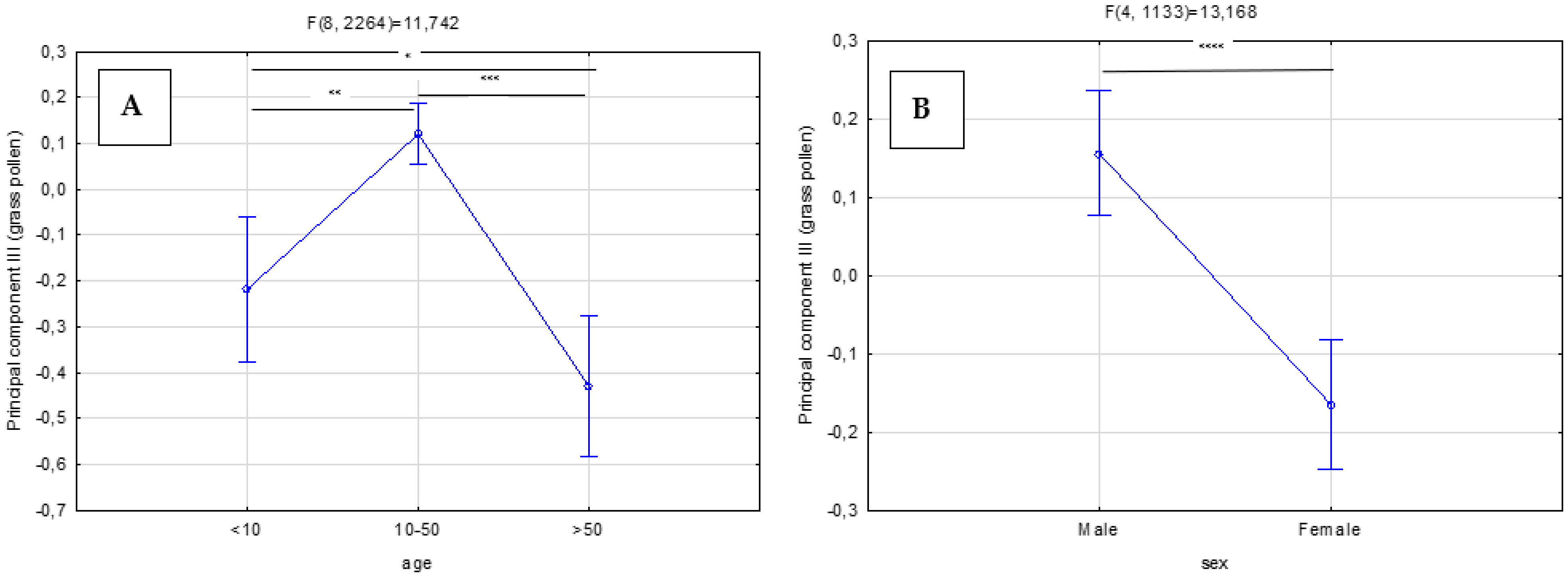

Figure S3. Comparison of variance in mean wheal size in SPT with grass pollen allergens between age groups (panel A) and sexes (panel B); * p=0.026; ** p=0.0004; *** p=0.00002; **** p=0.00000005

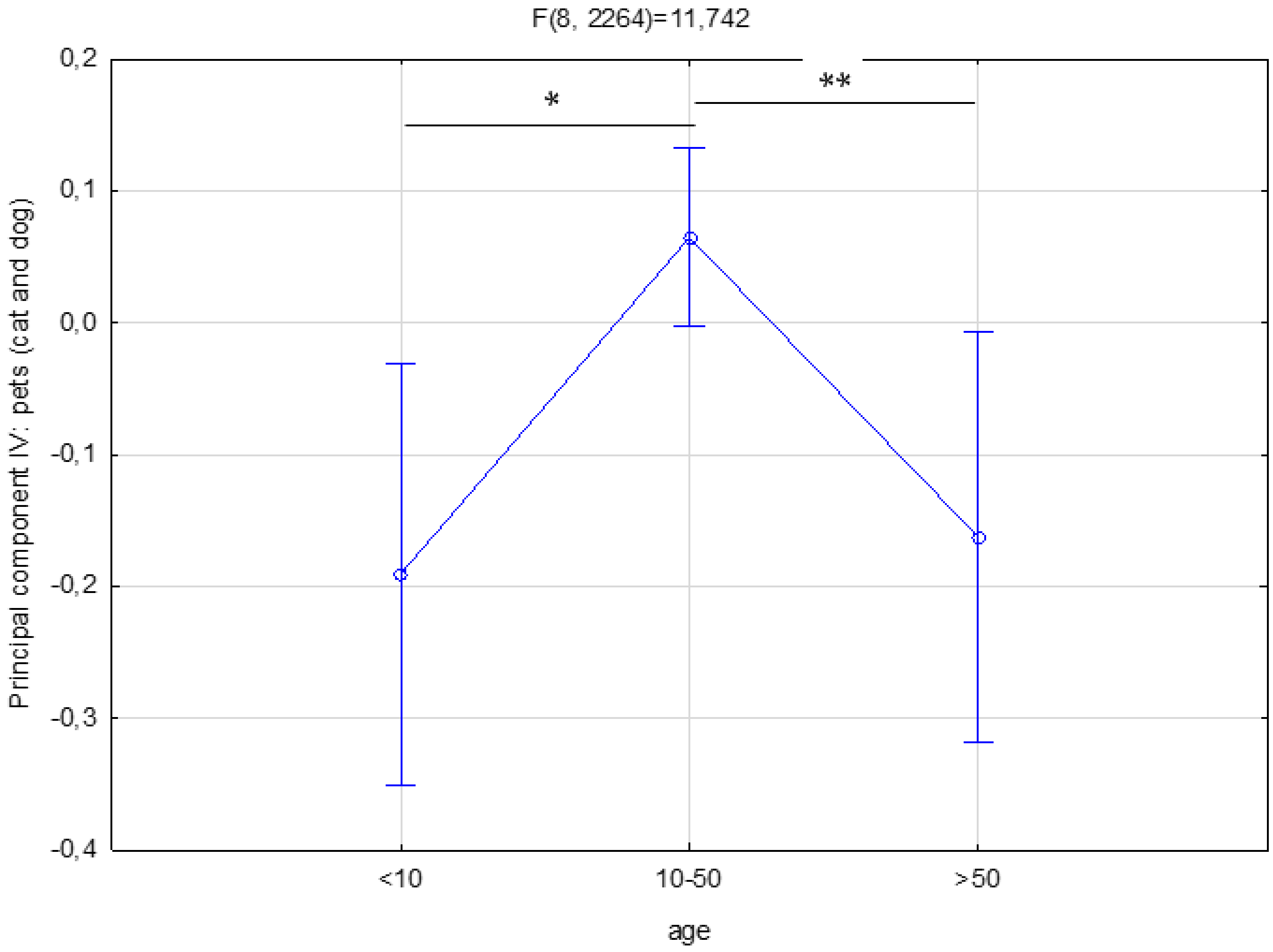

Figure S4. Comparison of variance in mean wheal size in SPT with cat and dog allergens (Factor IV) between age groups. *p=0.023; ** p=0.019

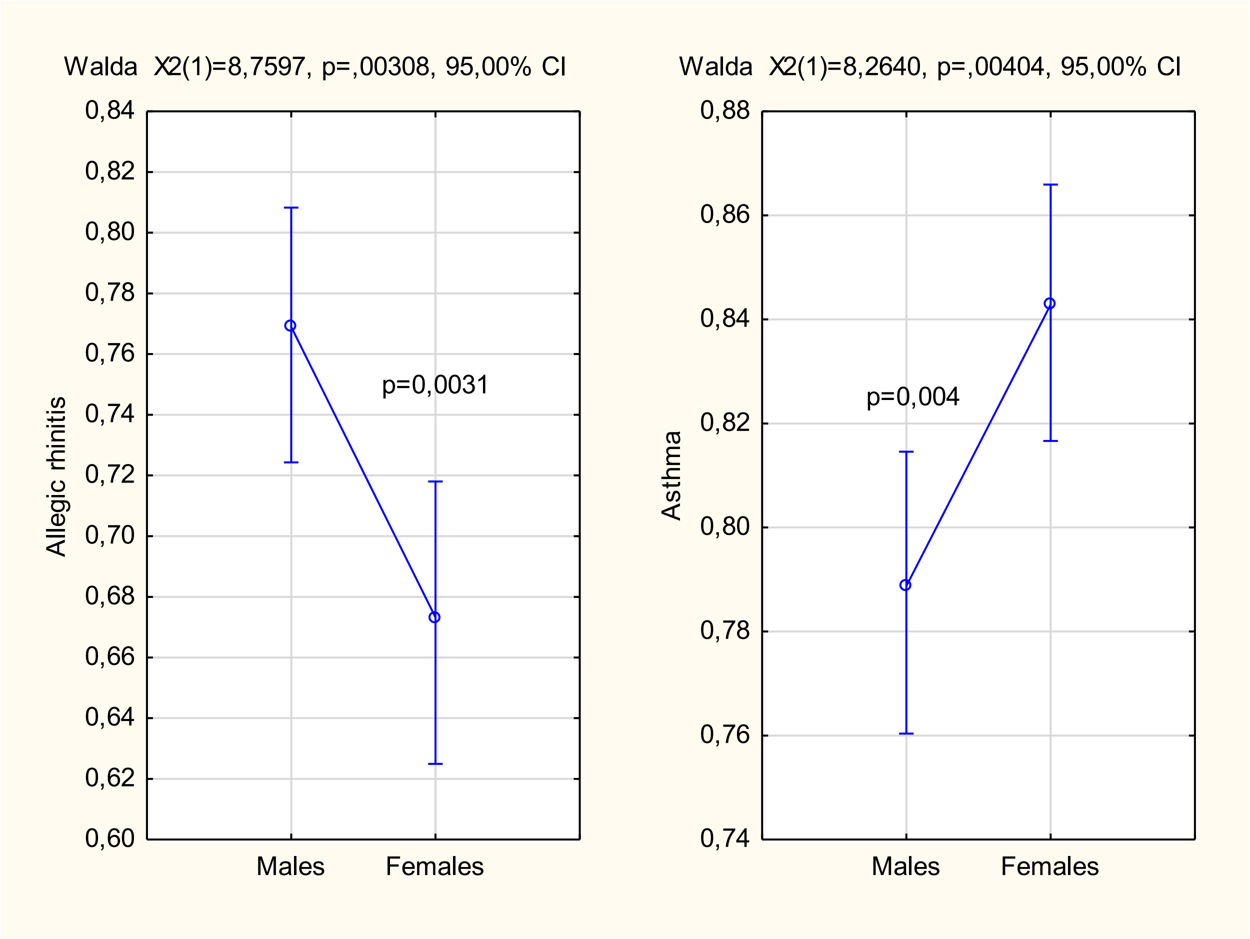

Figure S5: Differences between proportions in the prevalence of allergic rhinitis and asthma by sex categories of subjects.

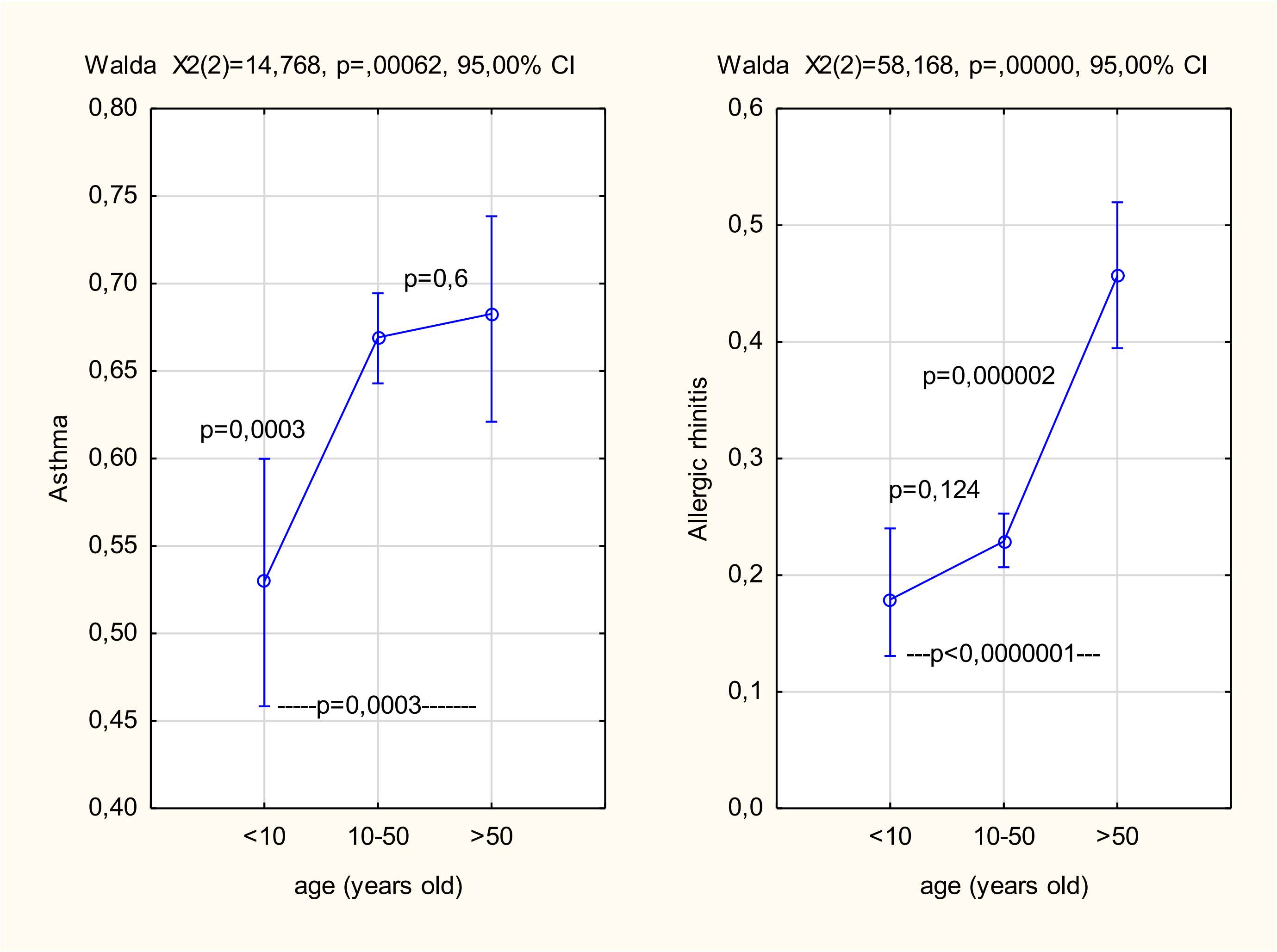

Figure S6. Differences between proportions in the prevalence of allergic rhinitis and asthma by age categories of subjects.

Table S1. Associations between physician-diagnosed allergic/atopic conditions (as retrieved from archival patients' charts) and allergic senitization ascertained by skin prick testing (SPT)

SPT	Allergic rhinitis	n	%	χ2 likehood test	*p-values corect	OR ±95% CI
1.	Grasses	863	67.74	109.56	0.000001	3.24(2.58-4.05)
2.	Rye	814	63.89	90.80	0.000001	2.92(2.33-3.65)
3.	Alder	584	47.25	44.58	<0.000001	2.22(1.75-2.82)
4.	Hazel	546	42.86	44.20	<0.000001	2.26(1.77-2.82)
5.	*Alternaria sp.*	228	19.52	14.68	<0.000001	1.96(1.38-2.77)
6.	Birch	648	50.86	24.83	0.000001	1.76(1.41-2.20)
7.	Cat	514	40.35	21.52	<0.000001	1.75(1.38-2.21)
8.	*Dermaophagoides farinae*	488	38.3	6.34	0.0121	1.35(1.07-1.70)
9.	*Dermaophagoides pteronyssinu*	561	44.03	10.35	0.0121	1.45(1.15-1.81)

SPT	Conjunctivitis					
1.	Alder	119	56.30	10.01	0.0025	1.82(1.25-2.65)
2.	Hazel	64	52.46	11.18	0.0025	1.86(1.29-2.70)
3.	Birch	70	57.38	5.31	0.0125	1.54(1.07-2.24)

SPT	Asthma					
1.	Dog	138	25.37	37.96	<0.000001	2.28(1.75-2.98)
2.	*Dermatophagoides farinae*	248	42.03	11.52	0.00072	1.43(1.16-1.75)
3.	*Dermaophagoides pteronyssinus*	284	48.14	14.91	0.00016	1.49 (1.21-1.82)
4.	Cat	281	47.63	42.16	<0.000001	1.96(1.60-2.41)

SPT	AZS Atopic eczema	n	%	χ2 likehood test	p-values corect	OR ±95% CI
1.	Birch	60	64.52	11.64	0.001	2.12(1.36-3.25)

2.	Alder	50	54.95	6.11	0.014	1.70(1.11-2.60)

SPT	Chronic inflammation of the nasal mucosa. nose and throat	n	%	χ2 likehood test	*p-values corect	OR ±95% Cl
1.	*Dermatophagoides farinae*	46	23.35	16.81	0.0001	0.49(0.35-0.69)
2.	*Dermatophagoides pteronyssinus*	52	26.40	21.68	0.000001	0.46(0.33-0.64)
3.	Alder	55	28.80	16.55	0.0001	0.51(0.37-0.71)
4.	Hazel	51	28.89	14.47	0.0002	0.52(0.38-0.74)
5.	Birch	67	34.01	15.87	0.0005	0.53(0.39-0.73)
6.	Grasses	99	50.25	9.76	0.00246	0.62(0.46-0.84)
7.	Rye	88	44.67	14.32	0.00028	0.56(0.42-64)
8.	Dog	17	9.29	9.3	0.00152	0.45(0.27-0.76)
9.	Cat	56	28.43	4.52	0.00602	0.59(0.36-0.96)
10.	*Alternaria*	24	12.18	14.75	0.0021	0.25(0.09-0.68)

SPT	Chronic sinusitis					
1.	*Dermatophagoides pteronyssinus*	26	29.89	5.33	0.023	0.58(0.36-0.92)
2.	Grasses	39	44.83	9.41	0.004	0.51(0.33-0.79)
3.	Rye	34	39.08	12.32	0.002	0.46(0.30-0.72)
4.	Cat	23	26.44	4.52	0.029	0.59(0.36-0.96)
5.	*Alternaria*	4	5.13	8.60	0.021	0.24(0.09-0.67)

SPT	Nasal polyps					
1.	Grasses	5	31.25	5.78	0.0035	0.29(0.10-0.84)
2.	Rye	4	25.0	6.85	0.0086	0.25(0.08-0.77)

SPT	Cough					
1.	Alder	14	27.45	4.87	0.036	0.50(0.27-0.94)
2.	Hazel	11	20.75	7.11	0.0322	0.41(0.21-0.81)
3.	Grasses	25	47.17	4.06	0.0466	0.57(0.33-0.99)
4.	Rye	22	41.51	5.51	0.0358	0.52(0.30-0.91)
5.	Cat	13	24.51	3.74	0.0466	0.54(0.29-1.02)

6.	*Alternaria sp.*	3	6.38	4.09	0.0357	0.32(0.097-1.0)

***** Benjamini-Hochberg corrects

Table S2. Gamma correlations for compared frequencies of positive SPT results (positive result defined as a wheal ≥3 mm diameter).

**Alder / Hazel pollen**	r=0,892	p<0,00001
**Alder /Birch pollen**	r=0,880	p<0,00001
**Birch / Hazel pollen**	r=0,858	p<0,00001
Alder/Mugwort pollen	r=0,254	**p=0,346**
Hazel / Mugwort pollen	r=0,299	**p=0,0367**
Birch / Mugwort pollen	r=0,245	**p=0,341**
